# Organization of *Plasmodium falciparum* spliceosomal core complex and role of arginine methylation in its assembly

**DOI:** 10.1186/1475-2875-12-333

**Published:** 2013-09-18

**Authors:** Manzar Hossain, Shweta Sharma, Reshma Korde, Shivani Kanodia, Monika Chugh, Khushboo Rawat, Pawan Malhotra

**Affiliations:** 1 International Centre for Genetic Engineering and Biotechnology, Aruna Asaf Ali Marg, New Delhi 110067, India; 2 Present address: Cold Spring Harbor Laboratory, Cold Spring Harbor, New York 11724, USA; 3 Present address: Department of Biological Sciences, Tata Institute of Fundamental Research, Homi Bhabha Road, Colaba, Mumbai 400 005, India

**Keywords:** *Plasmodium falciparum*, Spliceosome, Sm proteins, Survival motor neuron protein, Tudor domain, Arginine methylation

## Abstract

**Background:**

Splicing and alternate splicing are the two key biological processes that result in the generation of diverse transcript and protein isoforms in *Plasmodium falciparum* as well as in other eukaryotic organisms. Not much is known about the organization of splicing machinery and mechanisms in human malaria parasite. Present study reports the organization and assembly of *Plasmodium* spliceosome Sm core complex.

**Methods:**

Presence of all the seven *Plasmodium* Sm-like proteins in the intra-erythrocytic stages was assessed based on the protein(s) expression analysis using immuno-localization and western blotting. Localization/co-localization studies were performed by immunofluorescence analysis on thin parasite smear using laser scanning confocal microscope. Interaction studies were carried out using yeast two-hybrid analysis and validated by *in vitro* pull-down assays. PfPRMT5 (arginine methyl transferase) and PfSmD1 interaction analysis was performed by pull-down assays and the interacting proteins were identified by MALDI-TOF spectrometry.

**Results:**

PfSm proteins are expressed at asexual blood stages of the parasite and show nucleo-cytoplasmic localization. Protein-protein interaction studies showed that PfSm proteins form a heptameric complex, typical of spliceosome core complex as shown in humans. Interaction of PfSMN (survival of motor neuron, tudor domain containing protein) or PfTu-TSN (Tudor domain of Tudor Staphylococcal nuclease) with PfSmD1 proteins was found to be methylation dependent. Co-localization by immunofluorescence and co-immunoprecipitation studies suggested an association between PfPRMT5 and PfSmD1, indicating the role of arginine methylation in assembly of *Plasmodium* spliceosome complex.

**Conclusions:**

*Plasmodium* Sm-like proteins form a heptameric ring-like structure, although the arrangement of PfSm proteins slightly differs from human splicing machinery. The data shows the interaction of PfSMN with PfSmD1 and this interaction is found to be methylation dependent. PfPRMT5 probably exists as a part of methylosome complex that may function in the cytoplasmic assembly of Sm proteins at asexual blood stages of *P. falciparum.*

## Background

Significant information about *Plasmodium* genome and its transcriptome/proteome profiles have been obtained in recent years, however, to date little knowledge exists about the regulatory processes such as splicing and post-translational gene regulation in human malaria parasite. The pathogenic protozoan parasite of malaria, *Plasmodium falciparum* possesses introns in ~54% genes, but the splicing processes in malaria parasite are not well understood [1]. Splicing is a fundamental process present in most eukaryotic cells, including *Plasmodium* that excises introns from its precursor mRNA (pre-mRNA) to generate mature mRNA [2]. Pre-mRNA splicing is catalysed by the spliceosome, which consists of U1, U2, U4/U6 and U5 snRNAs and numerous splicing-related proteins [3]. In human and yeast, splicing process has been well understood and consists of a two-step, trans-esterification reaction accomplished by a macromolecular assembly called the spliceosomes [4-8].

The small nuclear ribonucleoprotein particles (snRNPs) are the major structural and functional components of spliceosome. Each snRNP is composed of specific snRNAs, several snRNP-specific proteins and Sm core proteins [9,10]. The small nuclear RNAs of the snRNPs play diverse roles in intron recognition as well as in splice site definition and are intimately involved in the spliceosomal catalysis. Most of the snRNPs contain a set of seven common, Sm proteins- B/B’, D1, D2, D3, E, F, G and a few other specific proteins that bestow specificity to the spliceosome function [11]. The evolutionary, conserved, human Sm proteins contain two Sm motifs and form a heptameric ring around a conserved nucleotide sequence motif (Pu AU_4-6_GPu) termed as ‘Sm site’ on the snRNAs, which is the structural hallmark of these particles [12]. Hence, the general architecture and biogenesis of spliceosomes appears to be similar among different organisms [13,14]. In addition to the canonical Sm proteins, other proteins carrying the Sm motifs have been identified in many eukaryotes. In yeast, nine such proteins exist that are designated as Lsm (Sm-like) proteins. Two different complexes have been described for Lsm proteins; Lsm 2–8 forms a complex with U6 snRNA and mediate interaction of U6 snRNA into the U4/U6.U5 tri-snRNP; whereas Lsm1-7 are involved in the decay of cytoplasmic mRNA [15-17]. Thus, Sm and Lsm proteins make up an evolutionary conserved family of snRNP-associated proteins in eukaryotes. Overall, the snRNP biogenesis and assembly is a complex but well-ordered, protein-assisted process that involves two interacting units, SMN-complex and methyltransferase 7complex (methylosome). In addition to SMN, a related protein TSN has recently been reported to participate in SnRNP assembly. SnRNAs associate with Sm proteins in cytoplasm and the assembled snRNPs are subsequently imported to the nucleus where pre-mRNA splicing occurs [18-20]. The SMN complex stringently recognizes snRNAs and the RNA-binding Sm proteins and facilitates snRNP assembly [21].

Much of the current knowledge on the spliceosomal components and its assembly has come from mammalian and yeast systems [22-24]. Among the protozoan parasites, the Sm protein complexes have been described in *Trypanosoma* and *Leptomonas*[25-30]. The first protozoan Sm protein was identified as a spliced leader (SL) RNP core protein in *Leptomonas collosoma*[28]. Later, the full set of seven Sm proteins was identified in *Trypanosoma brucei* and *Trypanosoma cruzi*. These proteins were demonstrated to form the canonical heptameric ring-like structure by protein-protein interaction studies. *Trypanosoma* Sm proteins; SmD1 and SmD3 show deviations from the Sm consensus as they lack C-terminal RG dipeptide repeats [29,31-33]. Francoeur *et al.* provided initial evidence for the presence of snRNPs in *P. falciparum*[34]. Later on other components of *P. falciparum* spliceosome core complex were identified, including the five snRNAs mainly by *in silico* analysis [35-37].

Recently, a number of studies have reported the functional characterization of few *P. falciparum* splicing related proteins; PfSRPK1 and PfPrp16 [38-40]. The present study describes the sequence analysis and molecular architecture of *P. falciparum* spliceosome core complex. Sequence analysis revealed few variations in *Plasmodium* Sm- and Lsm-like proteins in comparison to their human homologues that result in deviations in the PfSm core architecture. The interaction studies revealed the association between PfSMN with a spliceosome core protein, PfSmD1. Immunoprecipitation data shows the co-existence of a parasite-specific protein, PfPRMT5 with PfSmD1*.* These findings thus provide evidence for the methylation dependent assembly of *Plasmodium* spliceosomes.

## Methods

### Molecular cloning of Sm genes

Human Sm proteins; SmB/B’ (PF14_0146), SmD1 (PF11_0266), SmD2 (PFB0865w), SmD3 (PFI0475w), SmE (MAL13P1.253), SmF (PF11_0280) and SmG (MAL8P1.48) sequences were used to search the complete sequence database of *P. falciparum* (PlasmoDB). Seven candidate Sm gene sequences (see Additional file 1: Table S1), which were embedded in an open reading frame of appropriate length, in *P. falciparum* genome were selected for molecular cloning. Complementary or genomic DNA, as required, was used as template in PCR reactions. Sequence of primer sets used for cloning the full length Sm genes from *P. falciparum* cDNA is provided in Additional file 1: Table S1. PCR conditions were optimized for each gene. PCR products were cloned in pGEM-T vector (Promega) and several clones were sequenced for each of the Sm genes by automated DNA sequencing.

### Sequence comparison among PfSm proteins and their evolutionary relatedness

Protein sequence databases from several organisms were used to identify sequences similar to *P. falciparum* Sm and Lsm proteins. Sm (B, D1, D2, D3, E, F, G) coding sequences from human (*Homo sapiens*), yeast (*Saccharomyces cerevisiae*) and *Trypanosoma* (*T. brucei, T. cruzi*), were retrieved from NCBI database and were aligned with PfSm protein sequences obtained from PlasmoDB. Homology-based blast search also identified sequences similar to Lsm from *P. falciparum*. The phylogenetic tree was drawn using PhyloDraw, version 0.8 [41].

### Expression and purification of PfSMN and PfSm proteins in *Escherichia coli*

PfSMN (PFC1050w) and PfSm proteins (SmB, SmD1, SmD2, SmD3, SmE, SmF and SmG) were cloned in NcoI and BamHI sites of pET41a + vector and expressed as a GST- fusion protein in BL21 (DE3) *Escherichia coli* cells. Proteins were purified on a glutathione-sepharose column (Amersham) according to the protocol provided by the manufacturer. Briefly, 200 ml culture was induced at OD_600_ of 0.6 with 0.5 mM IPTG at 37°C for three hours. The culture was harvested and resuspended in 10 ml ice-cold PBS (140 mM NaCl, 2.7 mM KCl, 10 mM Na_2_HPO_4_, 1.8 mM KH_2_PO_4_, pH 7.3) containing complete proteinase inhibitor cocktail (Roche). Proteins were released from cell by sonication for 4 × 15 sec on ice. Triton X-100 was added to a final concentration of 1% (v/v) and the cell lysate was incubated at 4°C for 30 min with gentle mixing to aid solubilization of the fusion protein. The samples were centrifuged at 15,000 g for 10 min at 4°C and the supernatant were added to 500 μl bed volumes of pre-equilibrated glutathione-sepharose beads at 4°C for 30 min. The beads were washed several times with PBS, and the recombinant protein was eluted with 10 mM of reduced glutathione. SDS-PAGE analysis was done to analyse the presence of protein in each fraction. The fractions containing purified recombinant protein were pooled and dialyzed against PBS.

### Generation of anti-PfSm serum, immunobloting and immunofluorescence microscopy

Polyclonal anti-PfSmD1, -PfSmD2 and -PfSmD3 antibodies were raised in mice using purified GST fusion proteins. Anti-PfPRMT5 (PF13_0323) antibodies were raised against peptide, CILNNRVQTEEWKNV corresponding to 487–500 aa of PfPRMT5 [42]. The peptide was conjugated to maleimide activated *Megathura crenulata* keyhole limpet haemocyanin (mcKLH) (Pierce Biotechnology) reagent for raising the antibodies. For the western blot analysis, protein extract of parasites at asexual blood stages was used. Briefly, parasites were released from infected erythrocytes by saponin 0.1% (w/v) treatment. After washing with PBS, parasite pellets were lysed by four freeze/thaw cycles and the lysate was cleared by centrifugation at 14,000 g for 20 min. Parasite proteins were resolved on 12% SDS-PAGE and transferred onto nitrocellulose membrane. Immunoblotting was performed using anti-PfSm antibodies (1:1,000 dilution), followed by incubation with horseradish peroxidase-labelled anti-mouse IgG (Sigma).

Indirect immunofluorescence assay was performed on thin blood smears of 3D7 parasite culture by a procedure described earlier [43]. Briefly, the slides with blood smears were air-dried and fixed with an acetone-methanol (9:1) mixture at −20°C for 40 min. Slides were blocked in PBS-containing BSA 5% (w/v) for two hours at 37°C followed by incubation with the anti-PfSm sera (1:200 dilution) in blocking buffer for one hour at room temperature. Subsequently slides were washed with PBS and incubated with anti-mouse IgG labelled with Cy3 (Sigma). The slides were again washed twice with PBS and stained with DAPI (4’,6’-diamino-2’phenylindole) at a final concentration of 2 μg/ml. Later slides were washed extensively with PBS-Tween-20 0.05% (v/v) and once with PBS and mounted with an antifade solution (Biorad) to retard photobleaching. The slides were examined using Confocal microscope Nikon A1 with a 100X oil immersion objective.

### Yeast two-hybrid analysis

Protein-protein interactions were analysed using the MATCHMAKER-III two-hybrid system (Clontech) according to the manufacturer’s protocol. Briefly, full-length coding regions of PfSm proteins were subcloned into DNA binding domain vector pGBKT7 and activation domain vector pGADT7, resulting in pGBK-PfSmX, pGAD-PfSmX (where X = B, D1, D2, D3, E, F and G) plasmids respectively. Plasmid pairs were cotransformed into the yeast strain AH109 by the lithium acetate method and transformants were selected on minimal synthetic dropout (SD) medium lacking tryptophan and leucine at 30°C for three to four days. For specific protein-protein interactions, each transformant was replicated on SD medium lacking tryptophan, leucine, histidine, and adenine and allowed to grow for seven days at 30°C.

### *In vitro* binding assay (GST pull-down assay)

For GST pull-down assays, the GST-PfSm fusion proteins (SmB, SmD1, SmD2, SmD3, SmE, SmF and SmG) were bound to glutathione–sepharose 4B beads and the beads were washed four times with binding buffer (20 mM HEPES pH 7.9, 100 mM KCl, 2.5 mM MgCl_2_, 0.2 mM EDTA, 0.1% Triton X-100 (v/v), 1 mM DTT and protease inhibitor cocktail). Full-length PfSm genes cloned in the pGBKT7 plasmids were used to synthesize *in vitro*-translated proteins in presence of [^35^S]-methionine (Amersham) using the TNT-coupled reticulocyte lysate system (Promega) according to manufacturer’s protocol. For each reaction, 20 μl of beads (containing ~5 μg of GST-PfSm proteins or GST-PfSMN protein or GST protein alone) and 10 μl of [^35^S] -labelled Sm proteins, prepared as described above, were incubated in 300 μl of binding buffer for two hours at 4°C. Beads were washed five times with binding buffer, and the bound proteins were eluted in SDS sample buffer and analysed by SDS- PAGE followed by autoradiography.

For methylation inhibition assay, 0.3 mM AdOx (Sigma), an inhibitor of protein methyltransferases was added to the rabbit reticulocyte lysate prior to *in vitro* translation reaction. For the pull-down assay, ~6 μg of purified GST-PfSMN protein was incubated with 10 μl of [^35^S] -labelled GST-tagged PfSm proteins- SmD1, D2, D3 produced by *in vitro* translation system. The reaction mixture was incubated with 30 μl Ni-NTA beads (Qiagen) in 250 μl binding buffer [20 mM Tris–HCl pH 8.0, 150 mM NaCl, 0.5 mM β-ME, 0.5% NP-40 (v/v)] at 4°C for two hours. In the control reaction, recombinant protein was omitted. Subsequently, beads were washed four times with 0.8 ml of binding buffer. Bound proteins were eluted in SDS sample buffer, analysed by SDS-PAGE, and visualized by autoradiography.

### Immunoprecipitation assays

For SmD1 immunoprecipitation study, Direct IP Kit (Pierce) was used and experiment was performed as per the manufacturer’s instructions. Briefly, 10 μg anti mouse-PfSmD1 antibody was coupled to the aminolink plus coupling resin using 3 μl sodium cyanoborohydride in 200 μl reaction volume. Parasite antigens were immunoprecipitated by incubating 300 μl (~200 μg) pre-cleared parasite lysate with coupled antibody with gentle mixing at 4°C overnight. At the end of the incubation resin was washed and antigens were eluted. Control experiment was performed as above using pre-immune mice IgG. Precipitated antigens were analysed on SDS-PAGE and stained using coomassie.

### In-gel trypsin digestion and mass spectrometric identification of SmD1 immuno-precipitated parasite proteins

The gel bands corresponding to 60–80 kDa size, which were specifically precipitated by PfSmD1 antibody were excised and digested with trypsin using In-Gel Tryptic Digestion Kit (Pierce) according to the manufacturer’s instructions. Briefly, the gel pieces were washed thrice in deionized water, twice in 25 mM NH_4_HCO_3_, and twice in acetonitrile 50% (v/v). The gel pieces were shrunk using 100% acetonitrile, and proteins were reduced by addition of 0.1 M DTT followed by an incubation step at 56°C for 45 min. The washing procedure described above was repeated, and proteins were alkylated by adding 55 mM iodoacetamide and incubating for 30 min at room temperature in the dark. After an additional wash and shrinkage, 10 ng/ul trypsin in 25 mM NH_4_HCO_3_ sufficient to cover the gel pieces was added followed by incubation on ice for 20 min. When the gel pieces were completely rehydrated, any excess trypsin solution was removed and replaced by 25 mM NH_4_HCO_3_, and samples were incubated overnight at 37°C. The digestion was stopped by adding 10 μl of glacial acetic acid, and the supernatant containing the tryptic peptides was harvested. An extraction step was carried out to recover the peptides from the gel slices by adding 50% acetonitrile and incubating at room temperature for 30 min. The supernatant was harvested again and pooled. The pooled peptide extracts were desalted using C18 desalting column (Millipore) followed by drying of the peptide samples by speedvac and subjected to MALDI-TOF analysis using MALDI MS-ABI Sciex 5800 TOF/ TOF mass spectrometer.

### Molecular modelling of Tudor domain

Three-dimensional models were constructed for the putative Tudor domain containing protein, PfSMN. Molecular modelling for *P. falciparum* Tudor domain was performed using 3D-PSSM software [44] based on the sequence and NMR structure of the human SMN Tudor domain [45]. The results obtained by the server were further analysed using the program UCSF Chimera package version 1 [46].

### AdOX treatment and determination of IC_50_

Assay was performed on synchronized parasite culture having rings at 14–16 hours, 0.8% parasitemia and 2% haematocrit. AdOX prepared in 10% DMSO and diluted in complete medium, was added at the final concentrations ranging from 0 to 100 μM in 100 μl culture assay volume, seeded in a 96-well flat bottom plate. The culture was incubated at 37°C for 48 hours in an atmosphere of 5% CO_2_, 1% O_2_ and 94% N_2_. Morphology and growth of the culture were monitored at 24 and 48 hours by visualizing Giemsa stained parasite smears under light microscope.

For determination of IC_50_, 100 μl of SYBR Green I in lysis buffer (0.2 μl of SYBR Green I/ml of lysis buffer) was added into each well after end of 48 hours’ incubation and the contents were mixed until no visible erythrocyte sediment remained. After one hour of incubation in the dark at room temperature, fluorescence was measured with a fluorescence multiwell plate reader from Perkin Elmer with excitation and emission wavelength bands centred at 485 and 530 nm, respectively, and a gain setting equal to 50. By using the accompanying Cytofluor software, the background reading for an empty well was subtracted to yield fluorescence counts for analysis. The counts were plotted against the drug concentration and curve fitting by non-linear regression (sigmoidal dose–response/variable slope equation) was done to yield the drug concentration that produced 50% of the observed decline from the maximum counts in the drug-free control wells (IC_50_).

## Results

### Identification of *Plasmodium falciparum* spliceosome’s RNP core proteins

**Figure 1:**
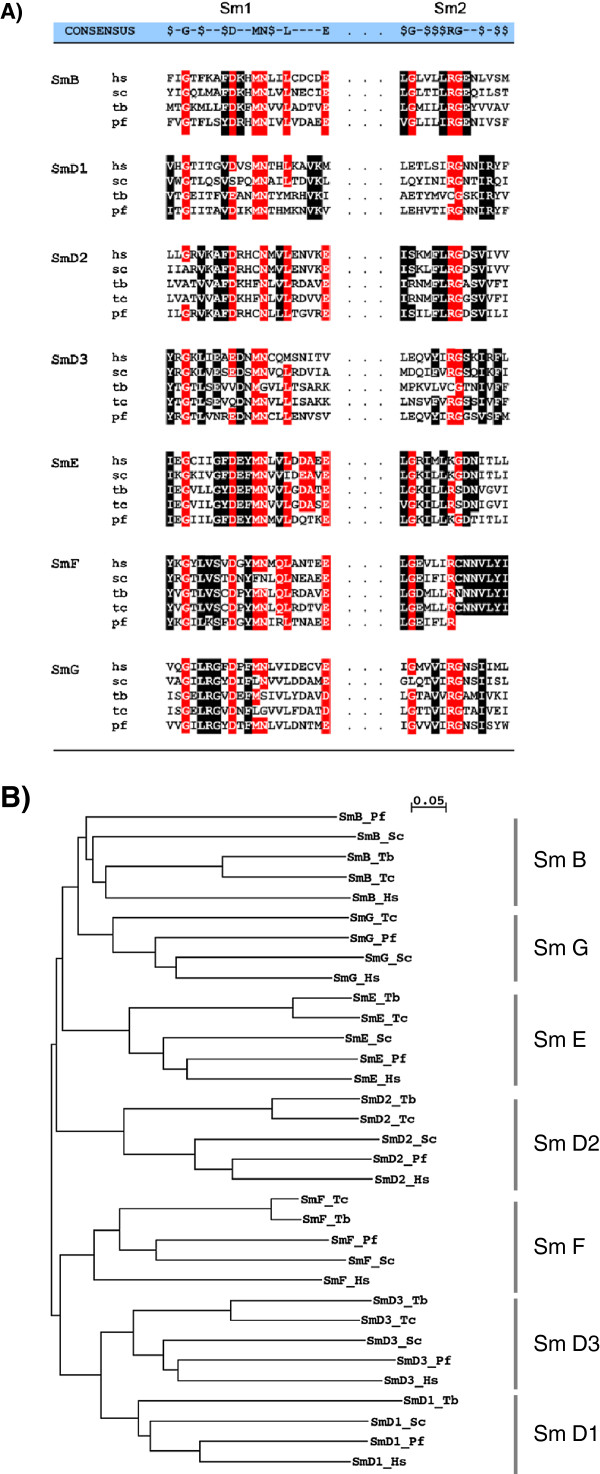
**Conservation of *Plasmodium falciparum *Sm proteins. (A)** Amino acid sequence alignment and comparison of Sm motifs 1 and 2 of *Plasmodium* Sm-B, D1, D2, D3, E, F and G core proteins with their human, yeast and *Trypanosoma* homologues. An alignment of the conserved Sm motifs 1 and 2 is presented with the consensus shown above ($, hydrophobic residue). Conserved amino acids are outlined by reverse print (red, overall conservation; black, subgroup conservation). **(B)** The unrooted phylogenetic trees of Sm proteins constructed with ClustalW using neighbour joining method. Abbreviations: Pf, *Plasmodium falciparum*; Hs, *Homo sapiens*; Tb, *Trypanosoma brucei*; Tc, *Trypanosoma cruzi* and Sc, *Saccharomyces cerevisiae*.

To know the existence of a *Plasmodium* spliceosome core complex, previously described sequences of *Plasmodium* Sm core proteins; SmB, SmD1, SmD2, SmD3, SmE, SmF, SmG and eight Lsm proteins (Lsm1-8) were aligned with their human, *T. cruzi, T. brucei* and *Sc. cerevisiae* homologues (Figure 1A and see Additional files 2, 3, 4: Figure S1-3). Sequence analysis showed that *P. falciparum* SmB, -D1, -D2, -D3, -F, and Sm-G proteins share 30, 51, 55, 39, 53, 31, and 48% identity with their human homologues and 14, 27, 29, 25, 33, 37, and 31% identity with trypanosome homologues, respectively. A phylogenetic tree was constructed to determine the evolutionary relationship of *Plasmodium* Sm proteins with the homologues in yeast, human and *Trypanosoma* (Figure 1B). The phylogenetic analysis revealed that *Plasmodium* Sm and Lsm proteins form a distinct subgroup with yeast and human Sm proteins, which is divergent from the *Trypanosoma* group (Figure 1B and see Additional file 2: Figure S1).

Alignment of PfSm and PfLsm sequences revealed that each of the PfSm and PfLsm sequences contain the bipartite Sm motifs with several conserved amino acid residues (Figure 1A, see Additional files 3 and 4: Figure S2-S3). Sequence comparisons among Sm homologues showed several differences in PfSm proteins from other eukaryotic Sm homologues. In comparison to human SmD1 protein, PfSmD1, possess only two arginine-glycine (RG) dipeptide repeats at the C-terminus (see Additional file 5: Table S2). Interestingly, these RG repeats are totally absent in TbSm homologues. The PfLsm4 contains a long stretch of RG repeats towards the C-terminal similar to the human Lsm4 (see Additional file 5: Table S2, and Additional 2: Figure S1), while these RG repeats are totally absent from TbLsm4 protein (see Additional file 5: Table S2). The *Plasmodium* Sm proteins also exhibit certain specific features which are absent in other homologues (Figure 1A). For example, the conserved DE/EA consensus sequence of Sm motif I of PfSmE is replaced by QT and DT instead of DN sequence was seen in Sm Motif 2 of the same protein. Most striking change is seen in Sm motif 2 of PfSmF protein. It lacks a conserved CNNVLYI sequence. These observations suggest that although *Plasmodium* PfSm and PfLsm proteins contain the conserved Sm motifs and other characteristic features, but certain subtle differences exist between the *Plasmodium* Sm and Lsm like proteins and their corresponding homologues.

### Expression and localization of *Plasmodium falciparum* Sm proteins

**Figure 2:**
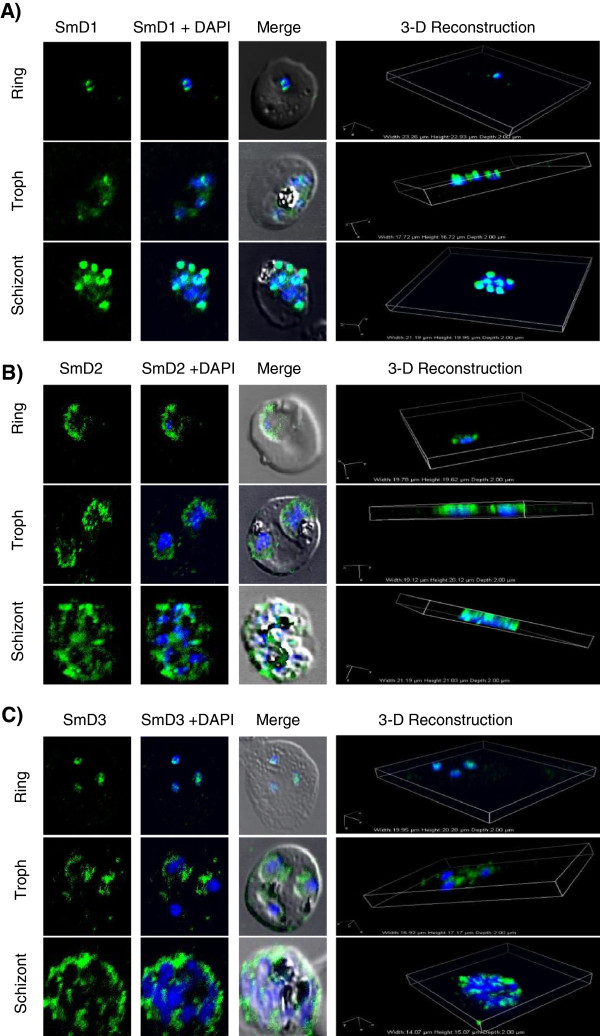
**Localization of PfSmD1, SmD2 and SmD3 at the asexual blood stages of *Plasmodium falciparum*.** Stage-specific expression of *Plasmodium* Sm proteins at asexual blood stages; ring (8 hr), trophozoite (36 hr) and schizont (42 hr) stages by immuno-localization. Immunofluorescence assay were performed using **(A)** anti mouse-PfSmD1, **(B)** anti- PfSmD2 and **(C)** anti-PfSmD3 antisera (1:50 dilution) on thin blood smears of *P. falciparum*. PfSm proteins showed nucleo-cytoplasmic localization.

RNA sequence and microarray analysis of *P. falciparum* have earlier shown that PfSm proteins are transcribed at asexual blood stages [37,47]. Protein expression and localization studies for the three PfSm proteins; PfSmD1,-D2 and -D3 were performed at the asexual blood stages by immunofluorescence assay using their respective antibodies raised against recombinant proteins in mice. As shown in Figure 2A-C, each of the three PfSm proteins are expressed at the three asexual blood stages; ring, trophozoite and schizont. These proteins showed nucleo-cytoplasmic distribution based on their partial co-localization with DAPI, a nuclear stain.

To confirm the expression of PfSm at asexual blood stages, western blot analysis was performed with the parasite lysate prepared from asynchronous asexual blood stages using mouse anti-PfSmD1,-D2 and -D3 antibodies. These antibodies specifically recognized the respective proteins in the parasite lysate, while the pre-immune serum failed to recognize any such protein in the same lysate (see Additional file 6: Figure S4). Together, immunofluorescence localization and western blot analysis showed that PfSm proteins are expressed at asexual blood stages of *P. falciparum.*

### Organization of the *Plasmodium falciparum* snRNP core complex

**Figure 3:**
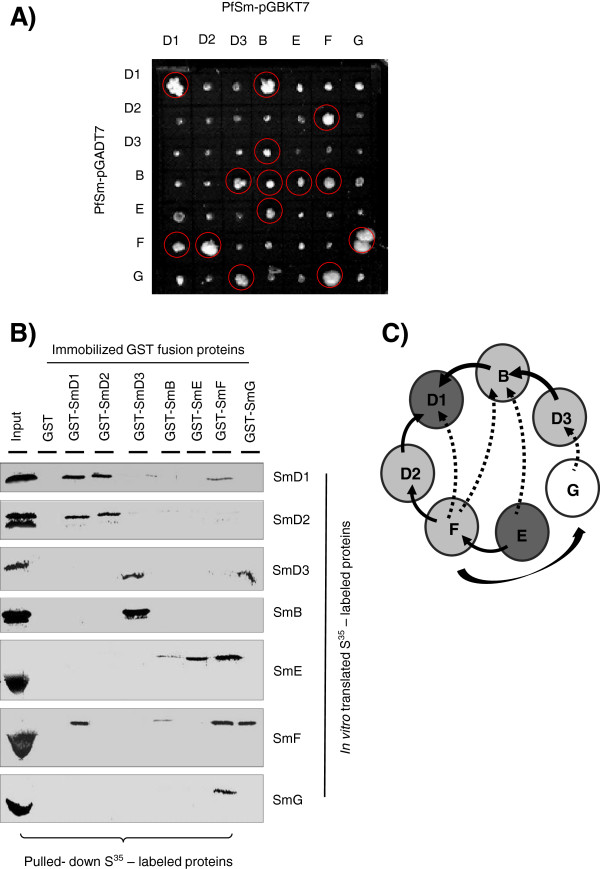
**Molecular interaction assays between *Plasmodium falciparum *Sm proteins. (A)** Yeast two-hybrid analysis to show interaction between seven *P. falciparum* Sm proteins. Cotransformants expressing PfSm proteins were selected on the minimal synthetic dropout medium lacking leucine, tryptophan, histidine, and adenine (hereafter termed SD/-Leu-Trp-His-Ade) at 30°C for three to five days. Positive interactions are encircled in red. **(B)***In vitro* interaction studies between seven *P. falciparum* Sm proteins. *In vitro*-translated, [^35^S] -labelled PfSm proteins (PfSmD1, -D2, -D3, -B, -E, -F, -G) were incubated with immobilized GST-PfSm proteins (lanes GST-SmD1, -D2, -D3, -B, -E, -F, -G) or, with GST alone (lane GST) as control. After washing, bound proteins were eluted and analysed by SDS-PAGE and fluorography. Input represents aliquots of radioactive proteins corresponding to 25% of that used in each of the binding reactions. **(C)** Proposed model of the *Plasmodium* Sm core complex. The specific heteromeric interactions between the *Plasmodium* Sm proteins are schematically depicted. Strong (−) and weak (−−) interactions among Sm proteins were indicated using black arrows. Homodimeric interactions are depicted with filled circles.

Considering the differences observed between *Plasmodium* and human Sm protein sequences, the organization of *P. falciparum* spliceosomal Sm core complex was investigated by performing protein-protein interaction studies among *Plasmodium* Sm proteins using yeast two-hybrid (Y2H) system and *in vitro* interaction studies. To perform Y2H analysis, each PfSm gene was subcloned into the pGADT7 as well as pGBKT7 vectors to generate prey and bait fusion proteins. The pairwise combinations of the recombinant plasmids, including the control vectors without insert, were introduced into yeast cells, AH109 and the transformants were grown in Trp^–^, Leu^–^, His^–^ and Ade^–^ selective plates. As shown in Figure 3A, 14 strong interactions among seven PfSm proteins, including the two homotypic interactions of D1/D1 and **B**/B and ten heterotypic interactions, D1/B, D2/F, D3/B, B/D3, B/E, B/F, E/B, F/D1, F/D2, F/G, G/D3, and G/F were observed. No interactions were observed when yeast cells were transformed with PfSm-pGADT7 vector and pGBKT7 vector alone or *vice versa*. As a negative control, yeast cells were transformed with empty prey (pGADT7) or bait vector (pGBKT7) and no cell growth was observed on the selective medium, confirming the specificity of interactions.

*In vitro* interaction assays were performed using the purified recombinant GST tagged PfSm proteins (see Additional file 6: Figure S4) and *in vitro* translated [^35^S] -labelled proteins. Interaction study using 7 X 7 pairwise combinations demonstrated strong and specific homodimeric and heterodimeric interactions between the individual Sm proteins (Figure 3B). The results showed the homodimeric interactions among PfSmD1, D2, D3, E and F proteins and heterodimeric interactions of PfSmD1 with PfSmD2 and PfSmF. In consensus with mammalian Sm core structure, the PfSmB showed a strong interaction with PfSmD3, while PfSmD3 apparently bound to itself as well as to PfSmG with similar efficiency. As expected, PfSmE showed an interaction with GST-PfSmF and *vice versa*. PfSmF showed an interaction with GST-PfSmG and *vice versa*, which is in contrast to human and yeast Sm core complex. SmB also showed weak interactions with SmE and SmF proteins.

Based on the combined results of Y2H and *in vitro* binding analysis, a model was built for the *Plasmodium* spliceosomal core complex (Figure 3C). Model suggested that the *P. falciparum* Sm proteins form a heptameric ring, however the organization of PfSm proteins in *Plasmodium* spliceosome complex appeared to vary from that of human or *Trypanosoma* core particles.

### PfSMN interact with the components of the spliceosome core complex and this interaction is methylation dependent

**Figure 4:**
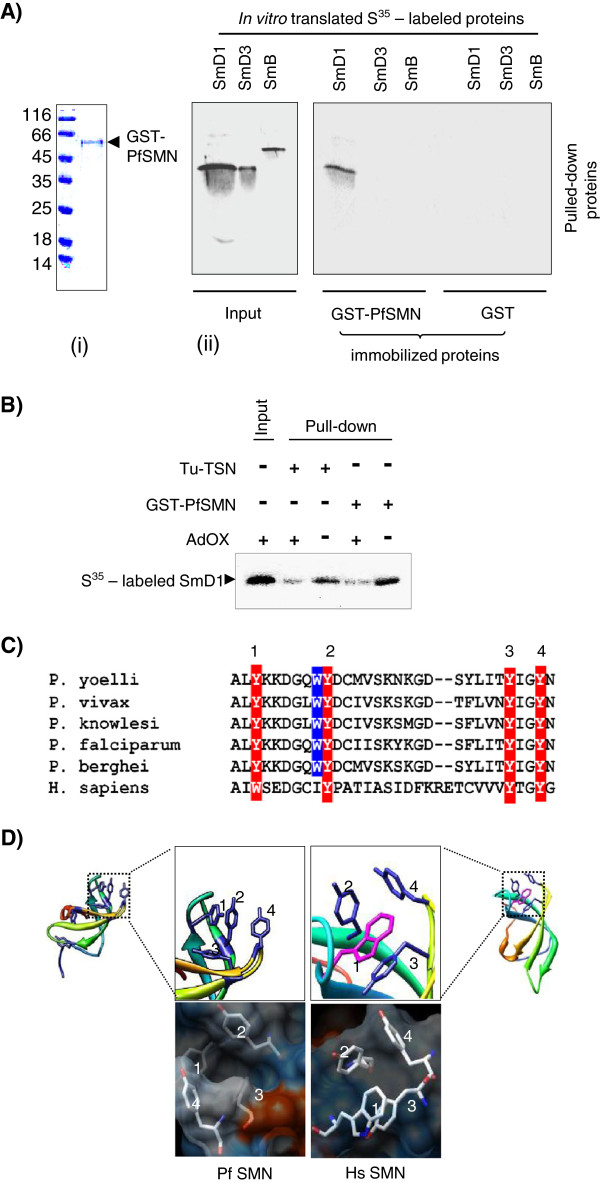
***Plasmodium *SMN protein interacts with PfSmD1 in methylation dependent manner. (A)** PfSMN interacts with PfSmD1 protein; (i) Coomassie stained SDS-PAGE gel of purified recombinant GST-PfSMN fusion protein; (ii) GST-PfSMN and GST were immobilized on glutathione sepharose beads. The immobilized proteins were incubated with *in vitro* translated, [^35^S] -labelled PfSmD1, PfSmD3 and PfSmB. Bound proteins were eluted by boiling in loading buffer, resolved by SDS-PAGE, visualized by autoradiography of the dried gel. 25% input of the [^35^S]-labelled proteins are shown. GST served as negative control. **(B)** Interaction of PfSMN and Tudor domain of PfTSN (PfTu-TSN) with PfSmD1 is methylation dependent. [^35^S]-methionine-labelled PfSmD1 protein was synthesized using the reticulocyte lysate system, in the presence or absence of the protein methyltransferase inhibitor, Adenosine periodates (AdOx) and incubated with either GST-PfSMN or PfTu-TSN. The interacting proteins were resolved on SDS-PAGE and detected by fluorography. **(C)** Sequence alignment of Tudor domain of PfSMN like protein with the Tudor domain of human hsSMN homologue. The well-conserved aromatic residues are outlined by reverse red print. **(D)** Molecular modelling of Tudor domain of PfSMN. Ribbon diagram (upper panel) as well as electrostatic potentials (lower panel) of the homology model of PfSMN compared with human SMN protein. The putative aromatic residues side chains are depicted in stick form. Similar to human SMN Tudor domain, the conserved aromatic residues of Tudor domain of PfSMN-like protein forms aromatic cage implicated in binding of methylated ligands.

Tudor domain-containing proteins (human SMN and TSN) have been shown to interact with snRNPs by interacting with Sm proteins to facilitate the spliceosome assembly [45]. An interaction between recombinant PfTu-TSN and PfSmD1 proteins has also been reported earlier [48]. To know the involvement of PfSMN protein with the *Plasmodium* spliceosome assembly, PfSMN protein was expressed and purified as a GST-fusion protein (Figure 4A (i)). The bacterially expressed PfSMN was incubated with *in vitro* translated [^35^S]-labelled PfSmB, PfSmD1 and PfSmD3 proteins. As shown in Figure 4A (ii), recombinant PfSMN interacted specifically with PfSmD1 and no interaction was observed for PfSMN with either PfSmB or PfSmD3 proteins.

In human spliceosomal UsnRNP assembly process, arginine methylation has been shown to be an important determinant for the interaction between the human SMN especially, its Tudor domain and Sm proteins [49]. Interaction studies were performed to investigate the role of methylation for the efficient interaction between the PfSMN or PfTu-TSN and PfSmD1 proteins. For the interaction analysis, [^35^S]-labelled PfSmD1 proteins were generated by *in vitro* translation using rabbit reticulocyte extract in the absence/presence of a methyltransferase inhibitor, adenosine periodate (AdOx). The methylated and unmethylated [^35^S]-labelled PfSmD1 proteins were allowed to interact with GST-tagged PfSMN or His-tagged PfTu-TSN proteins and the reaction mixtures were immunoprecipitated using mouse anti-GST and mouse anti-PfTu-TSN antibodies followed by analysis of bound PfSmD1 protein by autoradiography. As shown in Figure 4B, AdOx treatment considerably reduced the interaction of PfSmD1 with PfSMN or PfTu-TSN, therefore suggesting that the interaction between *Plasmodium* Tudor domain-containing proteins (PfSMN or PfTSN) and PfSmD1 is methylation dependent.

*In silico* modelling studies further supported the methylation dependent spliceosome interaction in *P. falciparum.* Homology modelling studies for Tudor domain of *Plasmodium* SMN protein revealed a rectangular aromatic cage-like structure made up of Tyr168, Tyr175, Tyr191 and Tyr194 residues (Figure 4C and D). Role of such an aromatic cage has been shown in human TSN protein (p100) and SMN proteins in the recognition and binding of the methyl group [50].

### Association of *Plasmodium* SmD1 with a parasite homologue of protein arginine methyltransferase 5 (PRMT5)

**Figure 5:**
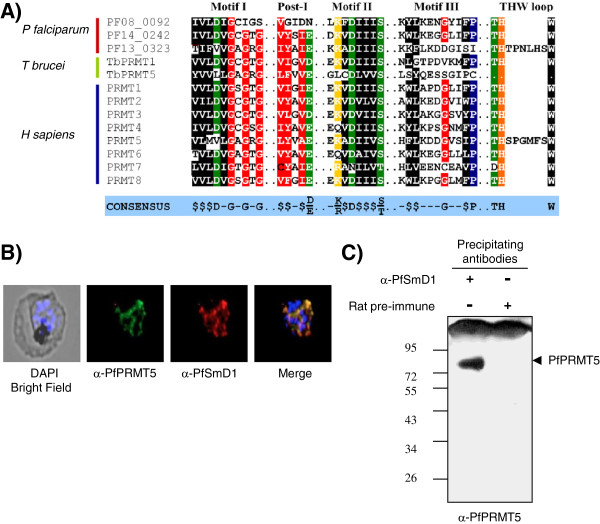
**Association of *Plasmodium *SmD1 protein with parasite protein arginine methyltransferase 5 (PRMT5). (A)** Amino acid sequence alignment of *Plasmodium* PRMTs methyltransferase motifs with corresponding *Trypanosoma* and human homologues. **(B)** Co-localization of PfSmD1 and PfPRMT5 proteins. Immunofluorescence assay was performed using mouse anti-PfSmD1 and rat anti-PfPRMT5 antisera (1:200 dilution) on thin blood smears of *P. falciparum*. **(C)** Immunoprecipitation of parasite proteins with mouse anti-PfSmD1 serum or control mouse pre-immune serum and probing with rat anti-PfPRMT5 antibodies in an immunoblot assay. Input shows the 25% of total parasite proteins used in an immunoprecipitation assay. Arrow indicates the precipitated PfPRMT5 protein band.

A protein complex referred as methylosome that contains Sm proteins, an arginine methyltransferase (JBP1/PRMT5), pICln and two other proteins has been implicated in the methylation of Sm proteins in Hela cells [51]. Based on the presence of conserved methyltransferase motifs, *Plasmodium* genome has been shown to encode three PRMTs, PRMT1 (PF14_0242), PRMT5 (PF13_0323) and PRMT8 (PF08_0092) (Figure 5A and see Additional file 7: Figure S5) [42]. To know the existence of a methylosome in *P. falciparum,* antibodies were raised against a peptide sequence corresponding to PfPRMT5 in rat, and immunolocalization studies were performed on the infected erythrocytes using mouse anti-PfSmD1 and rat anti-PfPRMT5 antibodies. As shown in Figure 5B, considerable co-localization was observed for both these proteins at asexual blood stages. To further confirm the association between these two proteins, parasite extract was immunoprecipitated with anti-PfSmD1 antibody or pre-immune serum and the precipitated samples were analysed for the presence of PfPRMT5 protein by western blot analysis using anti-PfPRMT5 antibody. As seen in Figure 5C, PfPRMT5 protein was detected in the anti-PfSmD1 antibody precipitated parasite extract, suggesting an association between PfPRMT5 and PfSmD1. To know whether the precipitated protein is actually PfPRMT5, the immunoprecipiated bands corresponding to ~60-80 kDa size were excised from the gel and subjected to in gel trypsin digestion followed by identification by mass spectrometry. As shown in Table 1, the immunoprecipitated band was truly the parasite PfPRMT5. In addition, peptides corresponding to s-adenosyl-l-homocysteine hydrolase (SAHH) protein were also identified, which has a role in regulation of intracellular methyltransferases in mammalian cell lines [52].

**Table 1:** PfSmD1 immunoprecipitated parasite proteins identified by MALDI-TOF spectrometry

**PlasmoDB accession**	**Identity**	**Unique peptide**	**MW (kDa)**	**Peptide matched**	**pI**	**SP/TM/ PEXEL**	**Maximum expression level (R/T/S)**
				LQWSTYISISK			
				MRIPIVIKK			
PF13_0323/PF3D7_1361000	arginine methyltran-sferase 5, putative	5	85.55	TKTNNNYYLK	6.84	No	R
				MYSYTKISQESSK			
				INKIQFLKK			
				KWTNIAKK			
PFE1050w/PF3D7_0520900	S-adenosyl-L-homocyste-ine hydrolase (SAHH)	3	53.84	GNKIIVLAR	5.71	No	R/T
				ICGYGDVGK			

**SP-** Signal peptide, **TM-** Transmembrane domain, **R-** ring, **T-** trophozoite, **S-** schizont.

### AdOx, a protein methylation inhibitor, inhibits parasite growth

To know the significance of arginine methylation in the malaria parasite development and growth, the effects of AdOx, an inhibitor of SAH hydrolase was analyzed on *in vitro* growth of 3D7 and Dd2 *P. falciparum* parasites. The effect of AdOx on parasite growth was quantified by determining IC_50_ value using SYBR green fluorescence method. AdOx inhibited the parasite growth with IC_50_ ~ 6.97 μM for 3D7 parasites and with IC_50_ ~ 40 μM for Dd2 parasites, a chloroquine- and mefloquine-resistant strain. AdOx-treated parasites also showed morphological abnormalities as indicated by appearance of ‘crisis forms’ in the treated cultures (see Additional file 8: Figure S6). Differential susceptibility of the two parasite strains towards AdOx is still to be investigated; however data presented here clearly demonstrated a role of arginine methylation in the growth and development of malaria parasite.

## Discussion

Splicing is a universal process present in all modern eukaryotic life and many of the components of splicing machinery seem to be conserved across the evolution [17]. A large number of studies performed in *Xenopus laevis* and HeLa cells have shown the role of two interacting complexes; SMN and PRMT-5 complexes in the assembly of Sm-class of snRNPs (UsnRNPs), essential unit of splicing machinery [51]. Compared to more evolved eukaryotes, knowledge about the architecture of the snRNP biogenesis and spliceosome complex is quite limited in lower eukaryotes [3,53]. In *P. falciparum* genome, ~7,406 introns have been predicted and splicing/alternate splicing appears to be the key processes that determine the mRNA and proteins generation [2]. In the present study, organization of the *P. falciparum* core-splicing machinery and the role of arginine methylation in spliceosome assembly were investigated.

### *Plasmodium falciparum* Sm core proteins are conserved and form a PfSnRNP core complex

Seven Sm-like protein, B/B’, -D1,-D2, -D3, -E, -F, and -G assemble onto UsRNPs to form the Sm core of the mammalian spliceosome snRNPs, U1, U2, U4/U6, and U5 [4]. In *P. falciparum,* a couple of *in silico* studies have previously identified putative homologues of human and yeast core splicing factors in *Plasmodium* genome [36,37]. However, a detailed sequence comparison analysis for *Plasmodium* spliceosome and spliceosome-related proteins with the human and *Trypanosoma* homologues has been lacking. In the present study, the results show that *Plasmodium* Sm-like as well as Lsm-like proteins possess the conserved Sm motifs 1 and 2 as well as the consensus amino acid residues [36,37]. Sequence comparison data showed few distinct features among *Plasmodium* Sm-like proteins in comparison to their human and *Trypanosoma* homologues*.* Significant variation was seen in PfSmF protein that lacked a conserved consensus sequence in Sm motif 2. Additionally, PfSm homologues contain fewer RG dipeptide repeats in comparison to human homologues; while such repeats are completely absent in *Trypanosoma* Sm proteins.

Given the fact that *Plasmodium* Sm proteins show few distinct features, the organization of Sm core proteins in *Plasmodium* was studied using *in vitro* interaction studies between recombinant PfSm proteins as well as by Y2H analysis. Similar approaches have been applied for understanding the organization of human, yeast and *Trypanosoma* snRNPs [10,15,16,24]. Strong interactions between SmD1-SmD1, SmB-SmD3, SmD3-SmG, SmF-SmB, and SmF-SmG were observed in both the interaction assays, suggesting the existence of a heptameric arrangement of SmD1-SmD2-SmE-SmF-SmG-SmD3-SmB/SmD1 as depicted in model in Figure 3C. This arrangement appeared to be slightly different than the heptameric arrangement shown for human, yeast and *Trypanosoma* Sm core structures [22-24,29].

### Interaction of PfSMN with PfSmD1 protein is methylation dependent

SMN and PRMT complexes have been shown to participate in the formation of splicing core complex in human [51,54,55]. Active SMN complex that brings Sm proteins to UsnRNAs. *In vitro* binding studies between human SMN and Sm proteins have provided evidence for direct and active involvement of human SMN/TSN in the spliceosome assembly [56]. Even the antibodies directed against tudor domain of human SMN have been shown to interfere with the UsnRNP assembly *in vivo*[32]. In human, Tu-TSN and Tudor domain of SMN have been shown to interact with symmetrically dimethylated SmB as well as SmD1/D3 proteins and these interactions are essential for the snRNP assembly [57]. An earlier report has described two Tudor domain -containing proteins; PfTSN and PfSMN in *P. falciparum*[48]. In the present study, their interactions with PfSm proteins were evaluated. *In vitro* binding studies showed an interaction between PfSmD1 and PfSMN proteins, indicating a role for the parasite’s Tudor domain-containing proteins in the *Plasmodium* spliceosome assembly. In case of HeLa cells, the SMN/TSN-Sm interactions have been shown to be methylation dependent; the methylation occuring at the arginine residues in RG rich motifs of Sm proteins [51]. The structural and functional dissection of the two tudor domain proteins in human; p100 and SMN have shown that the tudor domain forms an aromatic cage that recognizes and binds the methylated Sm proteins [45]. To know whether the methylation dependent interaction between PfTudor domain-containing proteins and PfSmD1 protein occurs in the malaria parasite, interaction studies between PfSMN or tudor domain of PfTSN and methylated or non-methylated PfSmD1 were performed. A strong interaction between PfSMN or PfTSN with methylated PfSmD1 suggested a methylation dependent spliceosome assembly mediated by tudor domain-containing proteins in *P. falciparum*. The homology modelling studies further supported the methylation dependent PfSMN/PfTSN interaction with PfSmD1 as the tudor domain of these proteins also form a rectangular aromatic cage-like structure (Figure 4D). It is pertinent to mention here that in *Drosophila* and *Trypanosoma,* Sm protein methylation is dispensable for snRNP assembly [30].

### PfSmD1 protein shows association with PfPRMT5

In human spliceosome core assembly, role of PRMT5-complex has been well established [51]. To know whether a similar complex is also involved in *Plasmodium* spliceosome asembly, co-localization and co-immunoprecipitation studies using anti-PfSmD1 and anti-PfPRMT5 antibodies were performed and the results clearly demonstrated an association between PfPRMT5 and PfSmD1. Association of PfPRMT5 with PfSm core assembly was further corroborated by mass analysis. In addition to PfPRMT5, an enzyme – SAHH was also identified in the immunoprecipitated lysate, which has been known to regulate s-adenosyl methyl-dependent, intracellular methylation reactions [52]. Together, these results showed the presence of a PRMT5-complex in *P. falciparum*, which is in accordance with the model proposed in human. It is important to mention that such a PRMT5-complex has not been reported in *Tetrahymena*, *Trypanosoma* and *Dictyostellium.* Using system level approach, a homologue of pICln protein was also identified, which has been shown to be a part of PRMT5-complex in human (see Additional file 9: Figure S2) [55]. Together, these results lend support to the methylation-dependent spliceosome assembly in the malaria parasite. These results are interesting as a methylation-dependent spliceosomal assembly has been reported to be absent in yeast as well as in *Trypanosoma.*

## Conclusions

The results of the present study suggest that the process of snRNP biogenesis in *Plasmodium* is quite similar to that of mammalian snRNPs biogenesis as *Plasmodium* Sm proteins also form a heptameric ring-like structure, although the arrangement of Sm proteins appears to be slightly different from human spliceosome core complex. Additionally, the data also shows that *Plasmodium* SmD1 protein exists in methylated form, which is required for its interaction with Tudor domain-containing proteins and this complex facilitates the assembly of the spliceosomal core complex. Further, the results show the presence of a PfPRMT5-complex which methylates the components of spliceosome core complex. The results thus provide insights into the snRNP assembly in *Plasmodium* and endow the opportunity to study the inimitability of parasite splicing process.

## Abbreviations

SMN: Survival motor neuron protein; PRMT: Protein arginine methyltransferase; Pf: *Plasmodium falciparum*; AdOx: Adenosine periodate; TSN: Tudor staphylococcal nuclease; Tu-TSN: Tudor domain of TSN.

## Competing interests

The authors declare that they have no competing interests.

## Authors’ contributions

MJH designed and carried out the experiments. RK helped MJH in cloning and protein expression. SS carried out MALDI/TOF experiment. SK, MC and KR provided data on immunoprecipitation and immunofluorescence. MJH, SS and PM analysed the data. MJH, SS, SK, MC and PM drafted the manuscript. All authors read and approved the final manuscript.

## Supplementary Material

Additional file 1: Table S1 Primer sequences designed and used in the present study. Description: The table provides all the primers used in the study. Click here for file

Additional file 2: Figure S1 *Plasmodium falciparum* Lsm proteins. Description: The data provided represent the phylogenetic analysis of Lsm proteins. Click here for file

Additional file 3: Figure S2 Multiple sequence alignment of Sm domains; Sm1 and Sm2 of Lsm proteins. Description: The data provided shows the alignment of Sm1 and Sm2 domains of Lsm proteins. Click here for file

Additional file 4: Figure S3 Multiple sequence alignment of full length Sm proteins. Description: The data provided represent the multiple sequence alignment of various full length Sm proteins with Human and Trypanosoma counterparts. Click here for file

Additional file 5: Table S2 In silico sequence analysis of spliceosome core proteins in *Plasmodium falciparum,* Trypanosoma and Human. Description: The table provides in silico sequence analysis of spliceosome core proteins in *Plasmodium falciparum*, Trypanosoma and Human. Click here for file

Additional file 6: Figure S4 Expression of Sm proteins. Description: The data provided represent the immunoblot, PCR ampilification and Coomassie stained gels of various Sm proteins. Click here for file

Additional file 7: Figure S5 Comparative analysis of Plasmodium and human PRMTs. Description: The figure represents various degree of homology between PRMTs. Click here for file

Additional file 8: Figure S6 Effect of adenosine periodate (AdOX) on intracellular growth of *P. falciparum*. Description: The data provided represent the effect of AdOX on parasite growth in two different parasite cell lines. Click here for file

Additional file 9: Figure S7 Identification of pICln in *Plasmodium falciparum* proteome database. Description: The data provided represent the alignment of pICln protein. Click here for file
