# Supplementary material for: Organization of Plasmodium falciparum spliceosomal core complex and role of arginine methylation in its assembly
Source: Malar J. 2013 Sep 18;12:333. doi: 10.1186/1475-2875-12-333 (PMC3848767; doi:10.1186/1475-2875-12-333)
Supplement: Additional file 1: Table S1 — Primer sequences designed and used in the present study. Description: The table provides all the primers used in the study. [file 1475-2875-12-333-S1.pdf]

Table S1. Primer sequences designed and used in the present study.
Oligonucleotide primer sequences used for PCR amplification of *PfSm, PfSMN, PfTSN* genes for the yeast two hybrid and protein expression.

PfTSN (PF11_0374)	PfTSN 6	GG***GGATCC***ATGAATGATTTAACAGCTTATGATAAT
	PfTSN 7	***CTCGAG***TCA***AAGCTT***ATTTTCTGTAGGTATTTTTAAA
PfSMN (PFC1050w)	SMN for	GG***GAATTCCATGG***ACGAATTTAATGAAACTTTA
	SMN rev	***GGATCC***TTCCTCATTATCATTGTAATCAAA
PfSmD1 (PF11_0266)	D1 For	GG***GAATCCATGG***AGATGAAACTAGTACATTTTTTG
	D1 Rev	***GGATCC***TCTTTTTGATAATTTTCTTCC
PfSmD2 (PFB0865w)	D2 For	GG***GAATTCCATGG***AGATGAAAAGTGAAGTTACCATA
	D2 Rev	***GGATCC***TTTAGGATTTCTTAAAATTAG
PfSmD3 (PFI0475w)	D3 For	GG***GGATCCATGG***CAGTTGGAATACCAATA
	D3 Rev	***GGATCC***TCATGCTTTTAAGTCTTTATTTTT
PfSmB (PF14_0146)	B For	GG***GAATTCCATGG***AGATGGGGAAAAATTCACGTTTAGA
	B Rev	***GGATCC***TTCTGCTGGAGGATTCACGTTAGG
PfSmE (MAL13P1.25 3)	E For	GG***GAATTCCATGG***CTACGACAAATAAAAAGTTA
	E Rev	***GGATCC***TGTTTCTTCATTTTTCACTTCCAT
PfSmF (PF11_0280)	F For	GG***GAATTCCATGG***AGATGAACCATTTATTTGGTATTGCT
	F Rev	***GGATCC***CCTTAAAAATATTTCACCAAGCGT
PfSmG (MAL8P1.48)	G For	GG***GAATTCCATGG***AGATGACTTTAACTGTTGGAAAGGCA
	G Rev	***GGATCC***TTTTATATTAACTTTGTCTAAACA
