# Supplementary material for: Organization of Plasmodium falciparum spliceosomal core complex and role of arginine methylation in its assembly
Source: Malar J. 2013 Sep 18;12:333. doi: 10.1186/1475-2875-12-333 (PMC3848767; doi:10.1186/1475-2875-12-333)

Fig. S1. *Plasmodium falciparum* Lsm proteins. (A) Genes corresponding to Lsm proteins of *Plasmodium falciparum* identified using BLAST search from PlasmoDB database, their size and estimated molecular mass of the PfLsm proteins are indicated. (B) Unrooted phylogenetic trees of Lsm proteins constructed using neighbor-joining method. Abbreviation used are Pf., *Plasmodium falciparum*; Hs., *Homo sapiens*; Tb., *Trypanosoma brucei*; Tc., *Trypanosoma cruzi* and Sc., *Saccharomyces cerevisiae*.

A)

Protein	Acc. No.	cDNA	MW (kDa)
Lsm1	PF11_0255	456	16.7 (152 AA)
Lsm2	PFE1020w	309	11.3 (103 AA)
Lsm3	PF08_0049	222	8.1 (74 AA)
Lsm4	PF11_0524	429	15.7 (143 AA)
Lsm5	PF14_0411	303	11.1 (101 AA)
Lsm6	PF13_0142	231	8.4 (77 AA)
Lsm7	PFL0460w	324	11.8 (108 AA)
Lsm8	MAL8P1.9	282	10.3 (94 AA)

B)

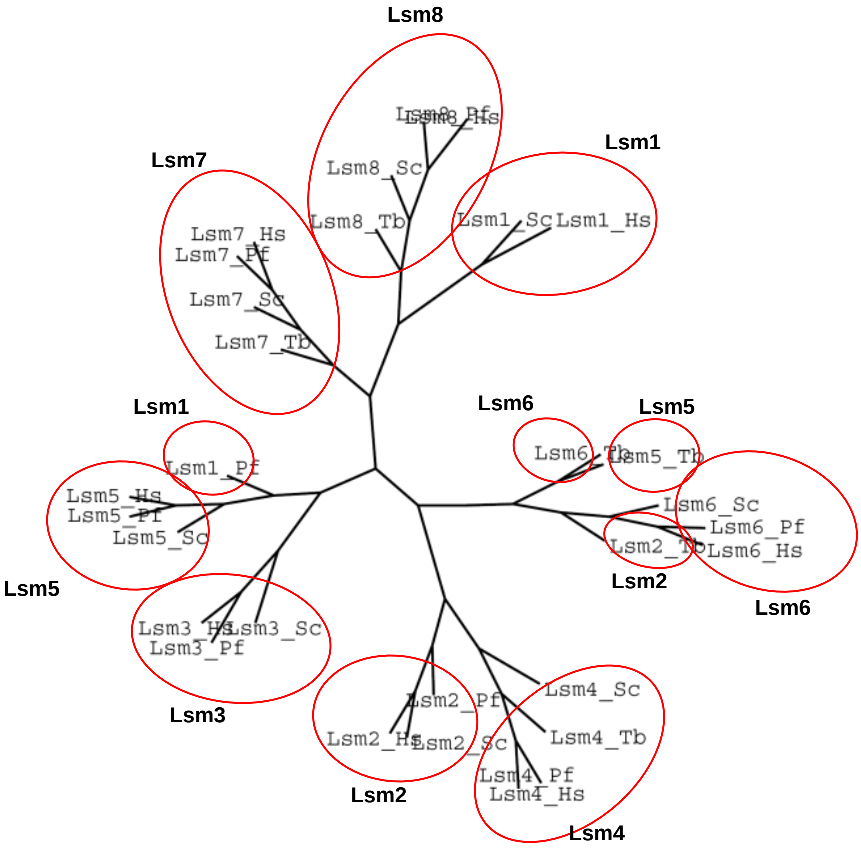

Supplement: Additional file 2: Figure S1 — Plasmodium falciparum Lsm proteins. Description: The data provided represent the phylogenetic analysis of Lsm proteins. [file 1475-2875-12-333-S2.pdf]
