# Supplementary material for: Organization of Plasmodium falciparum spliceosomal core complex and role of arginine methylation in its assembly
Source: Malar J. 2013 Sep 18;12:333. doi: 10.1186/1475-2875-12-333 (PMC3848767; doi:10.1186/1475-2875-12-333)

Fig. S2. Multiple sequence alignment of Sm domains; Sm1 and Sm2 of LSm proteins. Comparison of the eight *Plasmodium* Lsm proteins with their *Human* and *Yeast* homologues (hs, *Homo sapiens*; sc, *Saccharomyces cerevisiae*; Pf, *P. falciparum*). An alignment of the conserved Sm motifs 1 and 2 is presented with the consensus shown above ($, hydrophobic residue). Conserved amino acids are outlined by reverse print (red, overall conservation; black, subgroup conservation). Accession numbers: Human (Lsm1, NP_055277.1; Lsm2, AF182288_1; Lsm3, NP_055278.1; Lsm4, NP_036453.1; Lsm5, NP_036454.1; Lsm6, CAB45869.1; Lsm7, NP_057283.1; Lsm8, NP_057284.1.), *Plasmodium* (Lsm1, PF11_0255; Lsm2, PFE1020w; Lsm3, PF08_0049; Lsm4, PF11_0524; Lsm5, PF14_0411; Lsm6, PF13_0142.1; Lsm7, PFL0460w; Lsm8, MAL8P1.9) and Yeast (Lsm1, P47017; Lsm2, P38203; Lsm3, P57743; Lsm4, P40070; Lsm5, P40089; Lsm6, Q06406; Lsm7, P53905; Lsm8, P47093).

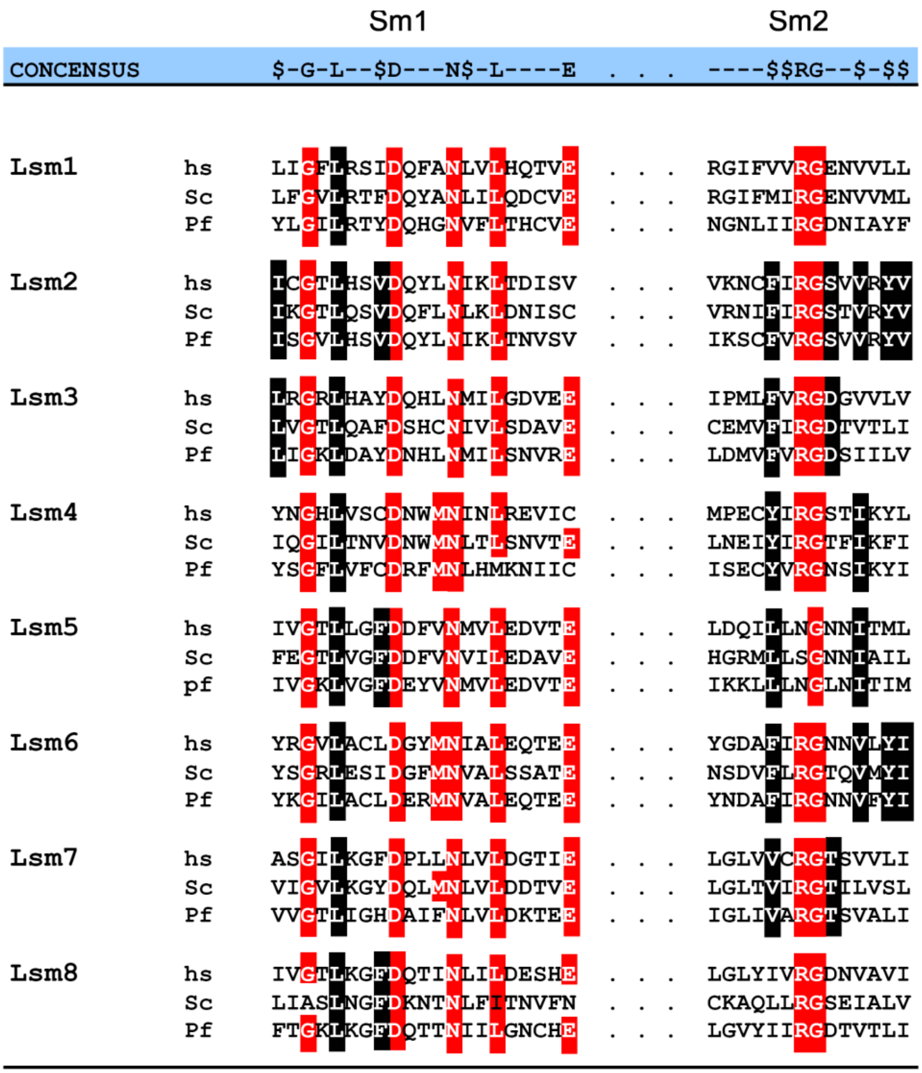

Supplement: Additional file 3: Figure S2 — Multiple sequence alignment of Sm domains; Sm1 and Sm2 of Lsm proteins. Description: The data provided shows the alignment of Sm1 and Sm2 domains of Lsm proteins. [file 1475-2875-12-333-S3.pdf]
