# Supplementary material for: Organization of Plasmodium falciparum spliceosomal core complex and role of arginine methylation in its assembly
Source: Malar J. 2013 Sep 18;12:333. doi: 10.1186/1475-2875-12-333 (PMC3848767; doi:10.1186/1475-2875-12-333)

Fig. S3. Multiple sequence alignment of full length Sm proteins. The alignment was performed using the Mac Vector program. The sequence of all the seven Sm proteins, of *Plasmodium* was aligned with Human and *Trypanosoma* counterparts. *Plasmodium* Sm proteins: SmB (PF14_0146), SmD1 (PF11_0266), SmD2 (PFB0865w), SmD3 (PFI0475w), SmE (MAL13P1.253), SmF (PF11_0280), and SmG (MAL8P1.48). *Trypanosoma brucei*: SmB (XP_340553.1), SmD1 (XP_823641.1), SmD2 (AAQ16042.1), SmD3 (XP_826797.1), SmE (XP_826840.1), SmF (XP_827316.1), and *T. cruzi* SmG (EAN96889.1). Human: SmB (P14678), SmD1 (P13641), SmD2 (P43330), SmD3 (P43331), SmE (P08578), SmF (X85372), and SmG (S55054). Yeast: SmB (P40018), SmD1 (Q02260), SmD2 (Q06217), SmD3 (P43321), SmE (Q12330), SmF (P54999), and SmG (P40204). The identical amino acids are shadowed box.

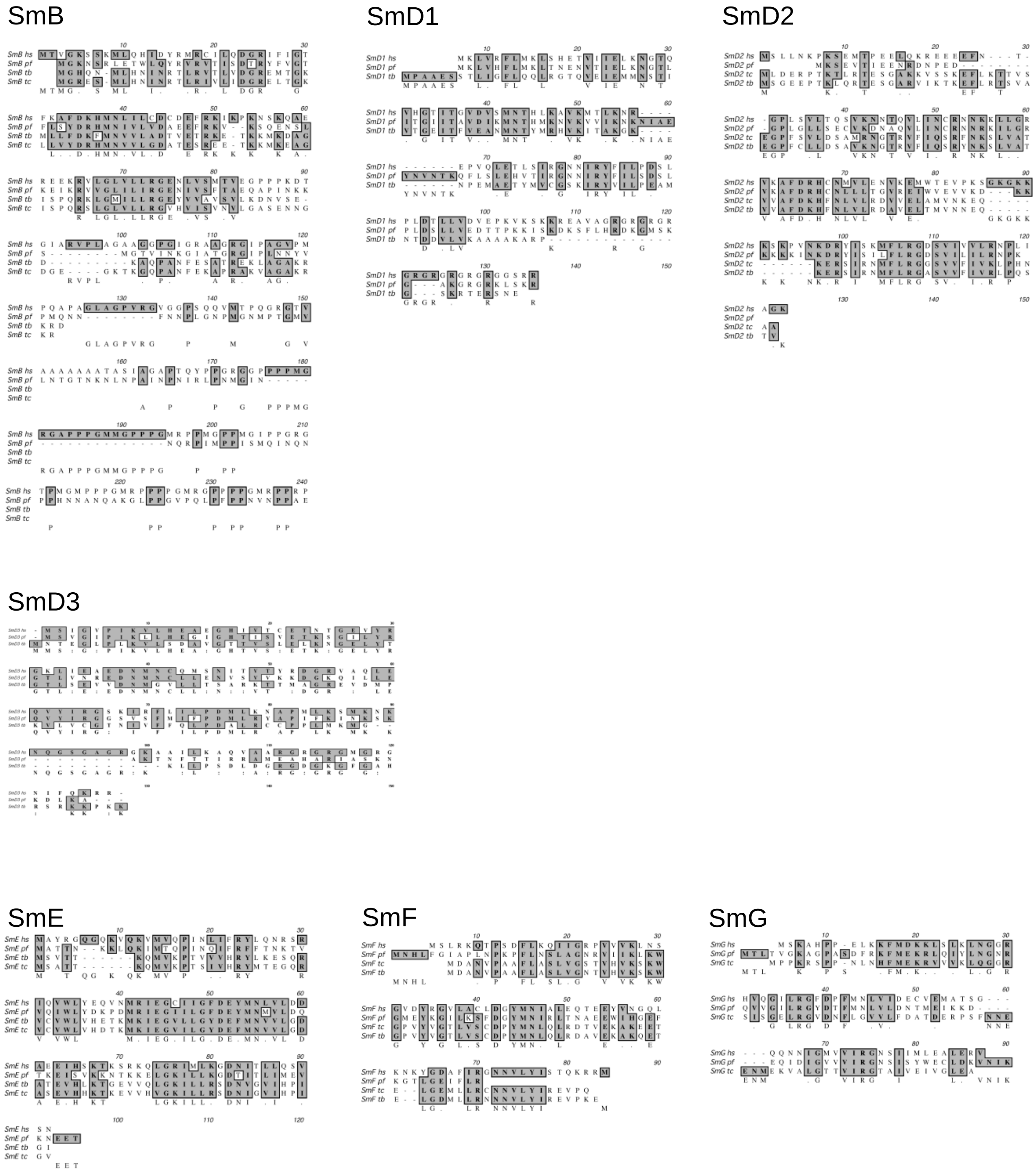

Supplement: Additional file 4: Figure S3 — Multiple sequence alignment of full length Sm proteins. Description: The data provided represent the multiple sequence alignment of various full length Sm proteins with Human and Trypanosoma counterparts. [file 1475-2875-12-333-S4.pdf]
