# Supplementary material for: Organization of Plasmodium falciparum spliceosomal core complex and role of arginine methylation in its assembly
Source: Malar J. 2013 Sep 18;12:333. doi: 10.1186/1475-2875-12-333 (PMC3848767; doi:10.1186/1475-2875-12-333)
Supplement: Additional file 5: Table S2 — In silico sequence analysis of spliceosome core proteins in Plasmodium falciparum, Trypanosoma and Human. Description: The table provides in silico sequence analysis of spliceosome core proteins in Plasmodium falciparum, Trypanosoma and Human. [file 1475-2875-12-333-S5.pdf]

Table S2. ***In silico* sequence analysis of spliceosome core proteins in *P. falciparum, Trypanosoma* and *Human*.**

'GR' dipeptide repeats are highlighted in red color.

Sm Proteins	*Trypanosome brucei*	*Plasmodium falciparum*	*Homo sapiens*
Sm D1	MPAAESSTLIGFLQQL**RG**TQVEIE<br>MMNSTIVTGEITFVEANMNTYMR<br>HVKITAKGKNPEMAETYMVCGS<br>KIRYVILPEAMNTDDVLVKAAAA<br>KARPGSKRTERSNE	MKLVHFLMKLTNENVTIELKNGTLIT<br>GIITAVDIKMNTHMKNVKVVIKNKNI<br>AEYNVNTKQFLSLEHVTI**RG**NNIRYFI<br>LSDSLPLDSLLVEDTTPKKISKDKSFL<br>HRDKGMSKGAK**GRGR**KLSKR	MKLVRFLMKLSHETVIIELKNGTQVH<br>GTITGVDVSMNTHLKAVKMTLKNRE<br>PVQLETLSI**RG**NNIRYFILPDSLPLDTL<br>LVDVEPKVKSKKREAVA**GRGRGRG**<br>**RGRGRGRGRGRGRGG**SRR
Sm D2	MSGEEPTKLQRTESGARVIKTKEF<br>LRTSVAEGPFCLLDSAVKNGTRV<br>FIQSRYNKSLVATVVAFDKHFNL<br>VLRDAVELTMVNNEQKERSIRN<br>MFL**RG**ASVVFIVRLPQSTV	MKSEVTIEENRDNPEDGPLGLLSECV<br>KDNAQVLINCRNNRKIL**GR**VKAFDR<br>HCNLLLTGVREIWVEVVKDKKKKKK<br>INKDRYISILFL**RG**DSVILILRNPK	MSLLNKPKSEMTPEELQKREEEEFNT<br>GPLSVLTQSVKNNTQVLINCRNNKKL<br>L**GR**VKAFDRHCNMVLENVKEMWTE<br>VPKSGKGKKKSKPVNKDRYISKMFL<br>**RG**DSVIVVLRNPLIAGK
Sm D3	MNTEGLPLKVLSDAVGTTVSLEL<br>KNGELYTGTLSEVVDNMGVLLTS<br>ARKTTMA**GR**EVDMPKVLVCGTN<br>IVFFQLPDALRCCPPLMKMGKLL<br>PSDLD**GR**GDGKGFGAHRSRKKP<br>KK	MSVGIPIKLLHEGIGHTISVETKSGILY<br>**RG**TLVNREKIKKRNFNEDNMNCLLE<br>NVSVVKKDGKQILLEQVYI**RGG**SVSF<br>MIFPDMLRYAPIFKINKSKAKTNFTTI<br>RRAMEAHARIASKNKDLKA	MSIGVPIKVLHEAEGHIVTCETNTGEV<br>Y**RG**KLIEAEDNMNCQMSNITVTYRD<br>**GR**VAQLEQVYI**RG**SKIRFLILPDMLK<br>NAPMLKSMKNKNQGSGA**GR**GKAAI<br>LKAQVAAR**GRGRG**M**GRG**NIFQKRR
Sm B	MGHQNMLHNINRTLRVTLVD**GR**<br>EMTGKMLLFDKFMNVVLADTVE<br>TRKETKKMKDAGISPQRKLGMIL<br>L**RG**EYVVAVSVLKDNVSEDKAQ<br>PANFESATREKLAGAKRKRD	MGKNSRLETWLQYRVRVTISDTRYF<br>VGTFLSYDRHMNIVLVDAEEFRKVKS<br>QENSLKEIKRVVGLILI**RG**ENIVSFTA<br>EQAPINKKSMGTVINKGIAT**GRG**IPL<br>NNYVPMQNNFNNPLGNPMGNMPTG<br>MVLNTGTNKNLNPAINPNIRLPNMGI<br>NNQRPIMPPISMQINQNPPHNNANQA<br>KGLPPGVPQLPFPPNVNPPAE	MTVGKSSKMLQHIDYRMRCILQD**GR**<br>IFIGTFKAFDKHMNLILCDCDEFRKIK<br>PKNSKQAEREEKRVLGLVLL**RG**ENL<br>VSMTVEGPPPKDTGIARVPLAGAAGG<br>PGI**GR**AA**GR**GIPAGVPMPQAPAGLA<br>GPV**RG**VGGPSQQVMTPQ**GRG**TVAA<br>AAAAATASIAGAPTQYPP**GRGG**PPPP<br>M**GRG**APPPGMMGPPPGMRPPMGPP<br>MGIPP**GRG**TPMGMPPPGMRPPPPGM<br>**RG**PPPPGMRPPRP
Sm E	MSVTTKQMVKPTVVVHRYLKES<br>QRVCVWLVHETKMKIEGVLLGY<br>DEFMNVVLGDATEVHLKTGEVV<br>QLGKILLRSDNVGVIHPIGI	MATTNKKLQKIMTQPINQIFRFFTNK<br>TVVQIWLYDKPDMRIEGIILGFDEYM<br>NMVLDQTKEISVKKNTKKELGKILLK<br>GDTITLIMEVKNEET	MAY**RG**QGQKVQKVMVQPINLIFRYL<br>QNRSRIQVWLYEQVNMRIEGCIIGFD<br>EYMNLVLDDAEEIHSKTKSRKQL**GR**I<br>MLKGDNITLLQSVSN
Sm F	MDANVPAAFLASLVGNTVHVKS<br>KWGPVYVGTLVSCDPYMNLQLR<br>DAVEKAKQETELGDMLLRNNNV<br>LYIREVPKE	MNHLFGIAPLNPKPFLNSLAGNRVIIK<br>LKWGMEYKGILKSFDGYMNIRLTNA<br>EEWIHGEFKGTLGEIFLR	MSLRKQTPSDFLKQII**GR**PVVVKLNS<br>GVDY**RG**VLACLDGYMNIALEQTEEY<br>VNGQLKNKYGDAFI**RG**NNVLYISTQ<br>KRRM
Sm G	MPPKRSPPNLNHFMEKRVVVKL<br>QG**GR**SISGEL**RG**VDNFLGVVLFD<br>ATDERPSFNNEENMEKVALGTTV<br>IRGTAIVEIVGLEA	MTLTVGKAGPASDFRKFMEKRLQIY<br>LNGNRQVVGIL**RG**YDTFMNLVLDNT<br>MEIKKDEQIDIGVVVI**RG**NSISYWECL<br>DKVNIK	MSKAHPPELKKFMDKKLSLKLN**GGR**<br>HVQGIL**RG**FDPFMNLVIDECVEMATS<br>GQQNNIGMVVI**RG**NSIIMLEALERV
Lsm 4	MSTKKSVIPLDVLRNC**RG**KVVSV<br>ELANGETINGTVMRVDRLMNLLL<br>KQCIRTGAEGDVFWKSRESLI**RG**<br>ASVRNVRMDERALVMPETRAAV<br>KNKSRPGTKKQATGGKKR**GR**GD	MKYGGKLLLYIKLLYTHLCFPLTLLK<br>CSQNQPVMVELKNGETYSGFLVFCD<br>RFMNLHMKNIICTSKDGDKFWKISEC<br>YV**RG**NSIKYIRVQDQAIEQAIEETAEQ<br>KARNM**GRGRGGRGRGRGG**MN**RG**<br>TY**GRGRGG**SMR**RGGRG**Q	MLPLSLLKTAQNHPMLVELKNGETY<br>NGHLVSCDNWMNINLREVICTSRDG<br>DKFWRMPECYI**RG**STIKYLRIPDEIID<br>MVKEEVVAK**GRGRGG**LQQQKQQK<br>**GRG**MGGA**GRG**VF**GGRGRGG**IPGT<br>**GRG**QPEKKP**GR**QAGKQ
