# Supplementary material for: Organization of Plasmodium falciparum spliceosomal core complex and role of arginine methylation in its assembly
Source: Malar J. 2013 Sep 18;12:333. doi: 10.1186/1475-2875-12-333 (PMC3848767; doi:10.1186/1475-2875-12-333)

Fig. S4 (A) Western blot analysis of asynchronous blood stages of *P. falciparum* parasite using anti PfSm-D1, -D2 and -D3 antibodies.(B) PCR amplification of the full length Sm genes from *Plasmodium falciparum* cDNA using gene specific primers listed in additional file 1(C) Coomassie stained SDS-PAGE of purified recombinant GST tagged Sm proteins. GST protein was run as a control. Molecular mass marker (Lane M). A low molecular weight band in GST-SmD1 & GST-SmD2 lanes is the degraded product of the corresponding protein.

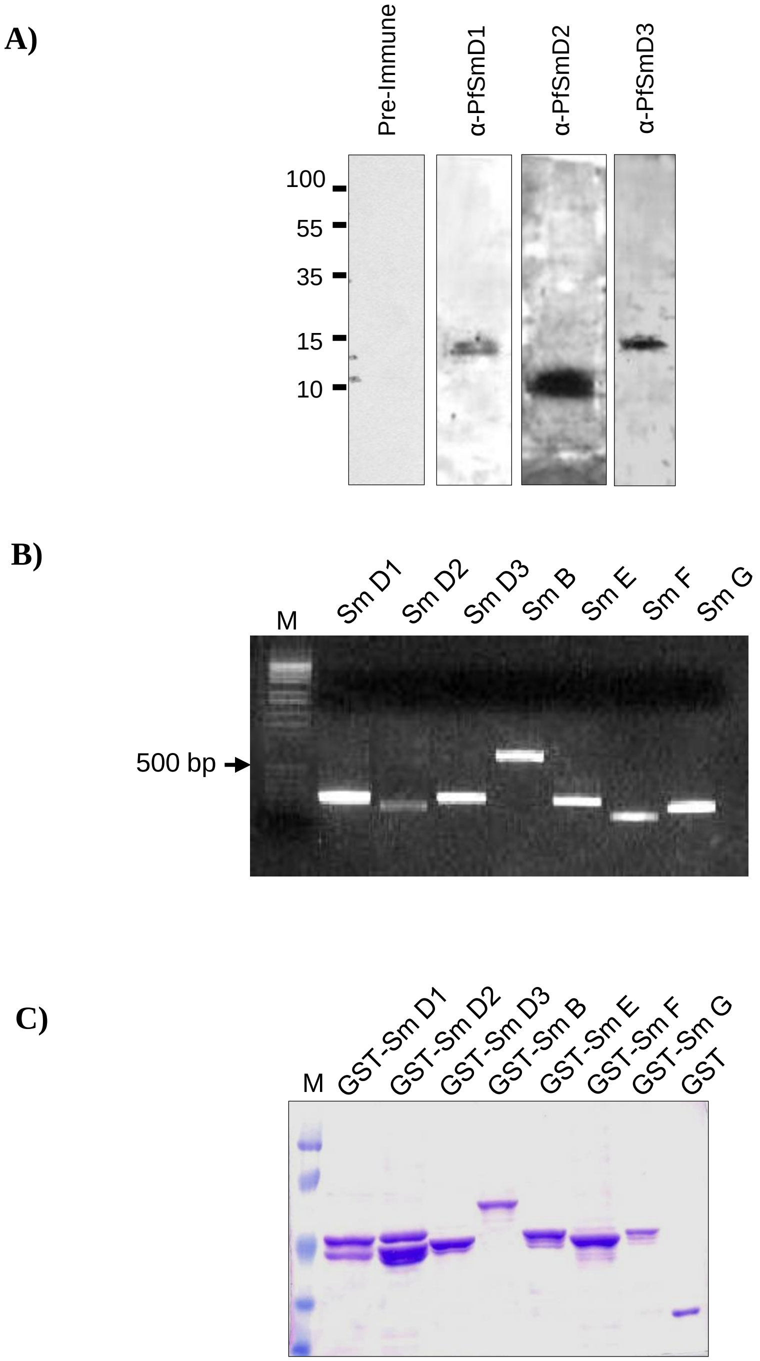

Supplement: Additional file 6: Figure S4 — Expression of Sm proteins. Description: The data provided represent the immunoblot, PCR ampilification and Coomassie stained gels of various Sm proteins. [file 1475-2875-12-333-S6.pdf]
