# Supplementary material for: Organization of Plasmodium falciparum spliceosomal core complex and role of arginine methylation in its assembly
Source: Malar J. 2013 Sep 18;12:333. doi: 10.1186/1475-2875-12-333 (PMC3848767; doi:10.1186/1475-2875-12-333)
Supplement: Additional file 7: Figure S5 — Comparative analysis of Plasmodium and human PRMTs. Description: The figure represents various degree of homology between PRMTs. [file 1475-2875-12-333-S7.pdf]

Fig. S5 Comparative analysis of *Plasmodium* and human PRMT's. *In silico* analysis of *Plasmodium* and human PRMT amino acid sequences showed various degree of homology. Numbers indicate the percent identity.

.

	PF08_0092 (912aa)	PF13_0323 (724aa)	PF14_0242 (401aa)
PRMT1 (361aa)	32	ND	49
PRMT2 (433)	32	ND	31
PRMT3 (531aa)	31	ND	42
PRMT4 (585aa)	25	23	34
PRMT5 (637aa)	ND	32	ND
PRMT6 (316aa)	29	32	35
PRMT7	ND	ND	22
PRMT8	31	ND	49
PRMT9	ND	ND	ND
