# Supplementary material for: Organization of Plasmodium falciparum spliceosomal core complex and role of arginine methylation in its assembly
Source: Malar J. 2013 Sep 18;12:333. doi: 10.1186/1475-2875-12-333 (PMC3848767; doi:10.1186/1475-2875-12-333)

Fig. S6 Effect of Adenosine periodate (AdOx) on intracellular growth of *P.falciparum*. Graph shows the IC_{50} curve of the AdOx treated parasites. Histogram represents the average mean of triplicates of parasite count. Bottom panel shows the morphology of control and AdOx treated (10 mm) 3D7 parasites after 48 hours.

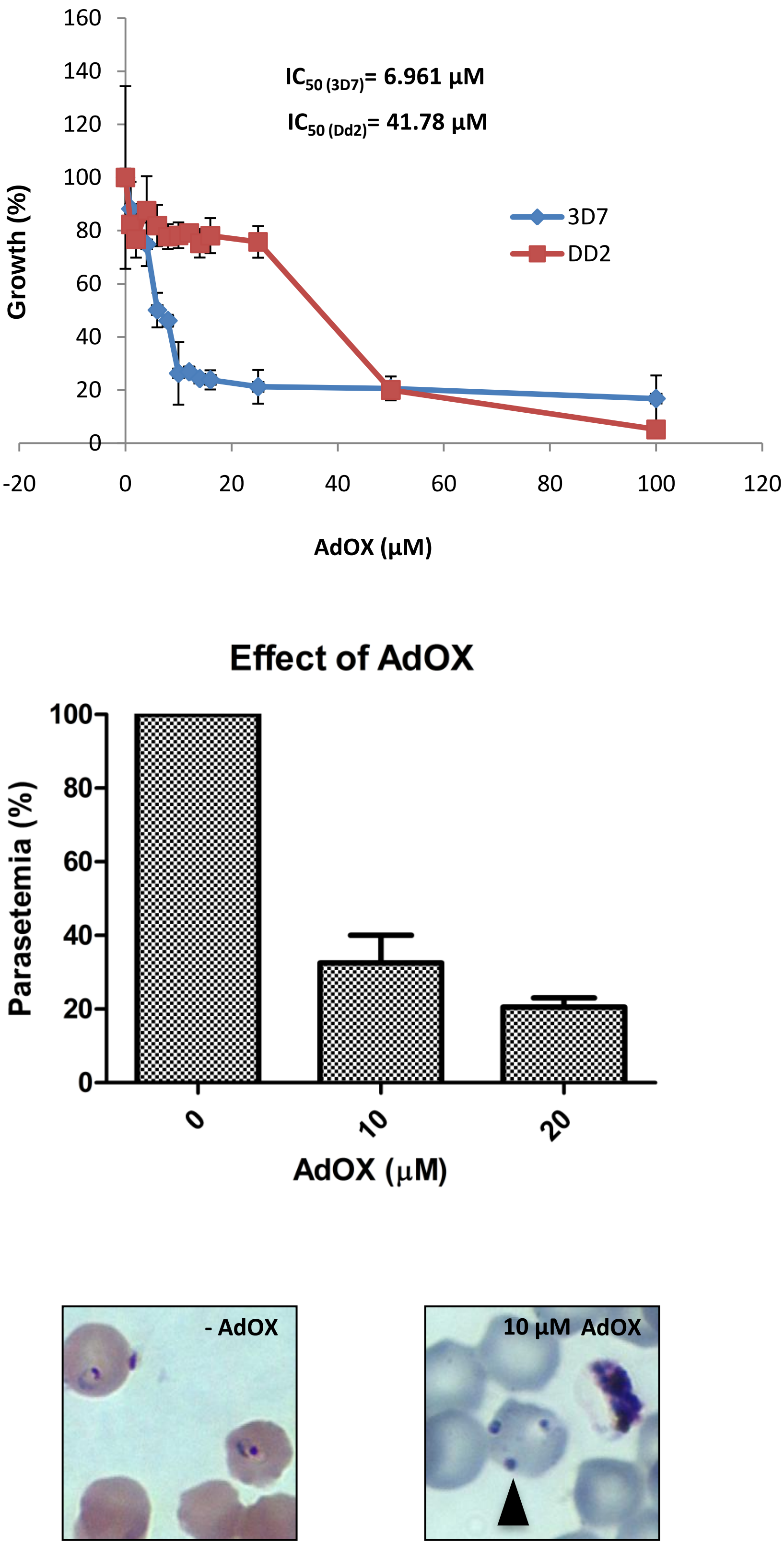

Supplement: Additional file 8: Figure S6 — Effect of adenosine periodate (AdOX) on intracellular growth of P. falciparum. Description: The data provided represent the effect of AdOX on parasite growth in two different parasite cell lines. [file 1475-2875-12-333-S8.pdf]
