# Supplementary material for: Organization of Plasmodium falciparum spliceosomal core complex and role of arginine methylation in its assembly
Source: Malar J. 2013 Sep 18;12:333. doi: 10.1186/1475-2875-12-333 (PMC3848767; doi:10.1186/1475-2875-12-333)
Supplement: Additional file 9: Figure S7 — Identification of pICln in Plasmodium falciparum proteome database. Description: The data provided represent the alignment of pICln protein. [file 1475-2875-12-333-S9.pdf]

Fig. S7 Identification of pICln in *Plasmodium falciparum* proteome database. Amino acid sequence alignment of putative PfpICln with that of human homolog.

```
H_sapiens         -MSFLKSFPPP-------GPAEGLLRQQPDTEAVLNGKGLGTGTLYIAESRLSWLDGSGL 52
P_falciparum      MPISLNSFKEEELISLEKGDFEPVLYKGSDIEFIYNKLNLGEGKLYILEKRLLWINEN-- 58
                      *:**          *  * :* : .* * : *  .** *.*** *.** *:: .

H_sapiens         GFSLEYPTISLHALSRDRSDCLGEHLYVMVNAKFEEESKEPVADEEEEDSDDDVEPITEF 112
P_falciparum      --ANKKNVTNFKELCTNNIYLN----HYEKNRNFYLHLLNEVNNISIDSSNIALHAITSD 112
                    : :  . .:: *. :.        :   * :*  .  : * : . :.*:  :..**.

H_sapiens         RFVPSDKSALEAMFTAMCECQALHPDPEDEDSDDYDGEEYDVEAHEQGQGDIPTFYTYEE 172
P_falciparum      KKICDNSCVYIQLNTDISDHLENWKNDEPVQRIIKSEQTNDDEENSDTDNDLPYDEISTP 172
                  : : .:...   : * :.:      : *  :    . :  * * :.: :.*:*

H_sapiens         GLSHLTAEGQATLERLEGMLSQSVSSQYNMAGVRTEDSIRDYEDGMEVDTTPTVAGQFED 232
P_falciparum      EILLVSKNSTDNDLIFRNMSNMDNSNNEDEEMDEEEDEQEEEEEEVEGDVVEDEENEDEE 232
                   :  :: :.  .   :..* . *.: :    . **. .: *: :* *..    .: *:

H_sapiens         ADVDH 237
P_falciparum      NK--- 234
                   .
```
